# Supplementary material for: Genome-Wide Identification of the Ferric Chelate Reductase (FRO) Gene Family in Peanut and Its Diploid Progenitors: Structure, Evolution, and Expression Profiles
Source: Plants (Basel). 2024 Jan 31;13(3):418. doi: 10.3390/plants13030418 (PMC10857631; doi:10.3390/plants13030418)
Supplement: Supplementary file 1 [file plants-13-00418-s001.zip › supplementary files/supplementary figures.pdf]

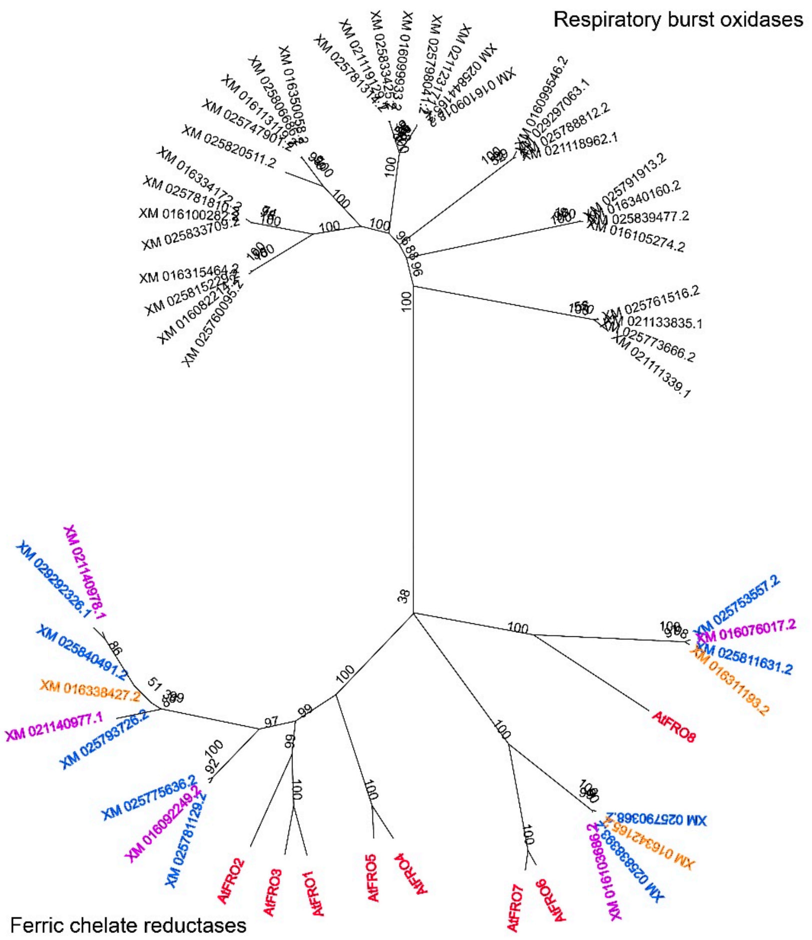

Figure S1. Screening FROs from candidate sequences identified by a BLASTP search against the peanut genome using phylogenetic analysis with AtFROs from *Arabidopsis*. A total of 9, 4, and 3 *FRO* genes were identified in peanut, *A. duranensis*, and *A. ipaensis*, respectively.

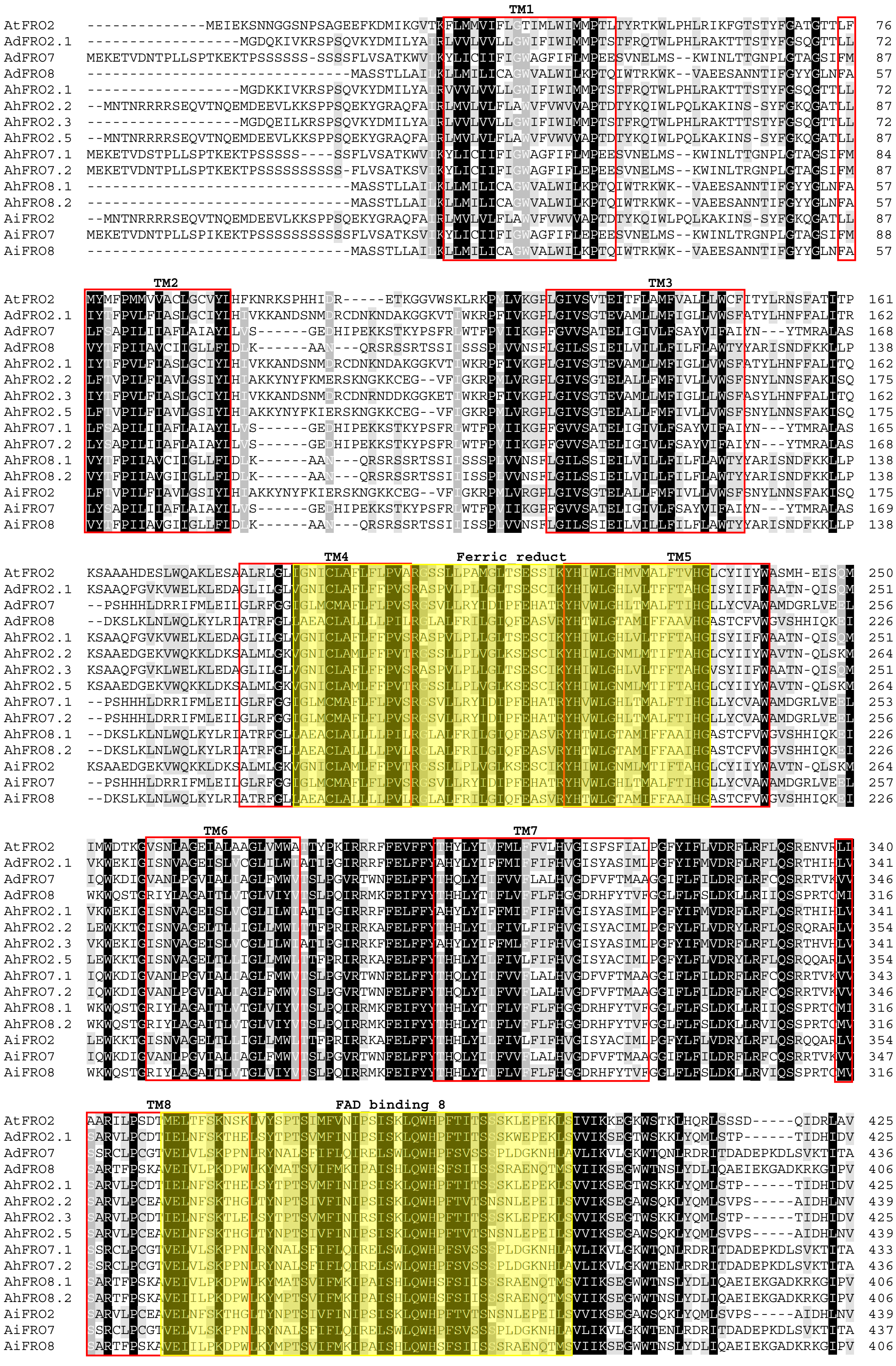
TM1
TM2
TM3
TM4
Ferric reduct
TM5
TM6
TM7
TM8
FAD binding 8
AtFRO2
AdFRO2.1
AdFRO7
AdFRO8
AhFRO2.1
AhFRO2.2
AhFRO2.3
AhFRO2.5
AhFRO7.1
AhFRO7.2
AhFRO8.1
AhFRO8.2
AiFRO2
AiFRO7
AiFRO8

```
                                              NAD binding_6 domains
AtFRO2      SVEGPYGPASADFLRHEALVMVCGGSGITPFISVIRDLIATSQKET-CKIPKITLICAFKKSSEISMLDLVLPLSGLETELSSDINIKIE  514
AdFRO2.1    SVEGPYGPASTNYLRHDSLVMVSGGSGITPFISIIRDLIYLSTTFK-YKTPKILLICAFKNTSSFSMLDLILPISGTPFEISN-LELQIE  513
AdFRO7      SVEGPYGHEVPYHLMYENLILVAGGIGLSPFLAILTDVLHRVREGKPCRPRNILVVWAVKKSNELPLLSSID-MESICPSFSDKVNINVD  525
AdFRO8      AIEGPYGPASLDFLKYDSLLLVAGGSGITPFLSILEELNSSSSKSR--YPSRIHLVYVIKKAQDFCLLHPIS-HLLVN-HSTENCHLNLK  492
AhFRO2.1    SVEGPYGPASTNYLRHDSLVMVSGGSGITPFISIIRDLIYLSTTFK-YKTPKILLICAFKNTSSLSMLDLILPISGTPFEISN-LELQIE  513
AhFRO2.2    HVEGPYGPVSTDYLRHETLVMISGGSGITPFVSIIRELIYLNITLR-YRTPKLTLICAFKNTSCLSMLDMILPSYGINGDISS-LQLQIE  527
AhFRO2.3    SVEGPYGPASTNYLRHDSLVMVSGGSGITPFISIIRDLIYLSTTFK-YKTPKILLICAFKNTSSLSMLDLILPISGTPFEISN-LELQIE  513
AhFRO2.5    HVEGPYGPVSTDYLRHETLVMISGGSGITPFVSIIRELIYLNITLR-YRTPKLTLICAFKNTSCLSMLDMILPCYGINGDISS-LQLQIE  527
AhFRO7.1    SVEGPYGHEVPYHLMYENLILVAGGIGLSPFLAILSDVLHRVREGKPCRPRNILVVWAVKKSNELPLLSSID-MESICPSFSDKVNINVD  522
AhFRO7.2    SVEGPYGHEVPYHLMYENLILVAGGIGLSPFLAILSDVLHRAREGKPCRPRNILVVWAVKKSNELPLLSSID-MESICPSFSDKVNINVD  525
AhFRO8.1    AIEGPYGPASLDFLKYDSLLLVAGGSGITPFLSILEELNSSSSKSR--YPSRIHLVYVIKKAQDFCLLHPIS-HLLVN-HSTENCHLNLK  492
AhFRO8.2    AIEGPYGPASLDFLKYDSLLLVAGGSGITPFLSILEELNSSSSKSR--YPSRIHLVYVIKKAQDFCLLHPIS-HLLVN-HSTENCHLNLK  492
AiFRO2      HVEGPYEPVSTDYLRHETLVMISGGSGITPFVSIIRELIYLNITLR-YRTPKLTLICAFKNTSCLSMLDMILPCYGINGDISS-LQLQIE  527
AiFRO7      SVEGPYGHEVPYHLMYENLILVAGGIGLSPFLAILSDVLHRAREGKPCRPRNILVVWAVKKSNELPLLSSID-MESICPSFSDKVNINVD  526
AiFRO8      AIEGPYGPASLDFLKYDSLLLVAGGSGITPFLSILEELNSSSSKSR--YPSRIHLVYVIKKAQDFCLLHPIS-HLLVN-HSTENCHLNLK  492
```

```
                                                                   TM9
AtFRO2      AFITRDND-AGDEAKAGKIKTLWFKPSLSDQSISSILGPNSWLWLGAILASSFLIFMIIIGIITRYYIYPIDHNTNKIYSLTSKTIIYIL  603
AdFRO2.1    AYITRDKE-FKSN-TLINLQTKWFKPNPNDAPIYAILGPNNWLWFGAIISSSFIIFLILIGIITRYYIFPKDHNTNGIFSYSLKALINML  601
AdFRO7      IYVTRESDPPLEEGHVYKPIKSSMCPMSSDFGMSVLVGTGDNFWSGLYVISSTVGFVILLVLLYIFYINPYGVYK----WWYKGLLFVIC  611
AdFRO8      LFVTQETQAGVGIKELLNEFSKVRTLELDTVCANYAVHGPESPSCLAAIVGMSSIIFLFFLICFNHVIIPSGKHS----NSSK--QKTPS  576
AhFRO2.1    AYITRDKE-FKSN-TLINLQTKWFKPNPNDAPIYAILGPNNWLWFGAIISSSFIIFLILIGIITRYYIFPKDHNTNGIFSYSLKALINML  601
AhFRO2.2    AYITREED-LKQNNSPIPLQSIWFKPNQKDSPISTILGPNSWVWLGAIISSSFIIFIILIGMINHYYIFPVDHNTNKIFSYPLKSSINML  616
AhFRO2.3    AYITRDKE-FKSN-TLINLQTKWFKPNPNDAPIYAILGPNNWLWFGAIISSSFIIFLILIGIITRYYIFPKDHNTNGIFSYSLKALINML  601
AhFRO2.5    AYITREED-HKQNNNSIPLQSIWFKPNQKDSPISTILGPNSWVWVGAIISSSFIIFIILIGMINHYYIFPVDHNTNKIFSYPLKSSINML  616
AhFRO7.1    IYVTRESDPPLEEGHVYKPIKSSMCPMSSDFGMSVLVGTGDNFWSGLYVISSTVGFVILLVLLYIFYINPYGVYK----WWYKGLLFVIC  608
AhFRO7.2    IYVTRESDPPLEEGHVYKPIKSSMCPMPSDFGMSVLVGTGDNFWSGLYVISSTVGFVILLVLLYIFYINPYGVYK----WWYKGLLFVIC  611
AhFRO8.1    LFVTQETQAGVGIKELLNEFSKVRTLELDTVCANYAVHGPESPCCLAAIVGMSSIIFLFFLICFNHVIIPSGKHS----NSSK--QKTPS  576
AhFRO8.2    LFVTQETQAGVGIKELLNEFSKVRTLQLDTVCANYAVHGPESPSCMAAIVGMSSIIFLIFLICFNHVIIPSGKHS----NSSK--QKAPS  576
AiFRO2      AYITREED-HKQNNNSIPLQSIWFKPNQKDSPISTILGPNSWVWVGAIISSSFIIFIILIGMINHYYIFPVDHNTNKIFSYPLKSSINML  616
AiFRO7      IYVTRESDPPLEEGHVYKPIKSSMCPMPSDFGMSVLVGTGDNFWSGLYVISSTVGFVILLVLLYIFYINPYGVYK----WWYKGLLFVIC  612
AiFRO8      LFVTQETQAGVGIKELLNEFSKVRTLQLDTVCANYAVHGPESPSCMAAIVGMSSIIFLIFLICFNHVIIPSGKHS----NSSK--QKAPS  576
```

```
                TM10
AtFRO2      VISVSIMATCSAAMLWNKKKYGKVESKQVQNVDRPSPTSSPTSSWGYNS-----LREIESTPQESLVQRTNLHFGERPNLKKLL----LD  684
AdFRO2.1    VMCVSIAIVASVGVLWNKKLSAKKGNNQVQHLEGLSPTVSPSSVVYYNN-GGNHNVELESLPSQLIAQATNVHYGERPDLRKIL----FE  686
AdFRO7      MVASVVIFGGFVVALWHFCEQRSSVKEKSNDSVKGDHKIEQNGFMAHKS---------SIQDS-SVAKSIVTRYGSRPNFKEIFEGMSEN  691
AdFRO8      WMVDLLLIAAFVLALA--CNGLVAILLRWRRLKKGIQPISEKDMKPLDL---------SSAEIRNALEEHEVHFGGRPNFEDIFGMFQEE  655
AhFRO2.1    VMCVSIAIVASVAVLWNKKLSAKKGNNQVQHLEGLSPTMSPSSVVYYNN-GGNHNVELESLPSQLIAQATNVHYGERPDLRKIL----FE  686
AhFRO2.2    VICVSIAMGASIAVLWNKKQNAKEAK-KVQNMEGSSPSVGSTNFKVCDI-----ERELESLPNQILANATNVHYAGRPDLRRLL----LE  696
AhFRO2.3    VMCVSIAIVASVGVLWNKKLSAKKGNNQVQHLEGLSPTVSPSSVVYYNNNGGNHNVELESLPSQLIAQATNVHYGERPDLRKIL----FE  687
AhFRO2.5    VICVSIAMGASAAVLWNKKQNVKEAK-KVQNMEGSSPSV---------------ERELESLPNQILANATNVHYGGRPDLRRLL----LE  686
AhFRO7.1    MVASVVIFGGFVVALWHFCEQRSSVKEKSNDSVKGDHKIEQNGFMAHKS---------SIQDS-SVAKSIVTRYGSRPNFKEIFEGMSEN  688
AhFRO7.2    MVASVVIFGGFVVALWHFCEQRSSVKEKSNDSVKGDHKIEQNGFMAHKS---------SIQDS-SVAKSIVTRYGSRPNFKEIFESMSEN  691
AhFRO8.1    WMVDLLLIAAFVLALA--CNGLVAVLLRWRRLKKGIQPISEKDMKPLDL---------SSAEIRNALEEHEVHFGGRPNFEDIFGMFQEE  655
AhFRO8.2    WIVDLLLIAAFVLALA--CNGLVAILLRWRRLKKGIQPISEKDMKPLDL---------SSAEIRNALEEHEVHFGGRPNFEDIFGMFQEE  655
AiFRO2      VICVSIAMGASAAVLWNKKQNVKEAK-KVQNMEGSSPSV---------------ERELESLPNQILANATNVHYGGRPDLRRLL----LE  686
AiFRO7      MVASVVIFGGFVVALWHFCEQRSSVKEKSNDSVKGDHKIEQNGFMAHKS---------SIQDS-SVAKSIVTRYGSRPNFKEIFESMSEN  692
AiFRO8      WIVDLLLIAAFVLALA--CNGLVAILLRWRRLKKGIQPISEKDMKPLDL---------SSAEIRNALEEHEVHFGGRPNFEDIFGMFQEE  655
```

```
AtFRO2      VEGSSVGVLVCGPKKMRQKVAEICSSGL-----------AENLHFESISFSW-  725
AdFRO2.1    IKGSSVGVFASGPKKMRQEVAAICSTGL-----------TENLHFESISFSW*  727
AdFRO7      WGHVDVGVLVCGPPTLQESVANEIRAHS-----MIRQRHYPIFHFHSHSFDL*  738
AdFRO8      TCGSNIGVLVCGPETLKESVAAACRKQSNCFKFGGGKRTEPCFTFHSLNFTL*  707
AhFRO2.1    IKGSSVGVFASGPKKMRQEVAAICSTGL-----------TENLHFESISFSW*  727
AhFRO2.2    LKGSNAGVLVSGPKEMRQEVASVCSSNL-----------AENLHFESFSFNW*  737
AhFRO2.3    IKGSSVGVFASGPKKMRQEVAAICSTGL-----------TENLHFESISFSW*  728
AhFRO2.5    LKGSNAGVLVSGPKEMRQEVAAVCSSNL-----------AENLHFESFSFNW*  727
AhFRO7.1    WGHVDVGVLVCGPPTLQESVANEIRAHS-----MIRQRHYPIFHFHSHSFDL*  735
AhFRO7.2    WGHVDVGVLVCGPPTLQESVANEIRAHS-----MIRQRHYPIFHFHSHSFDL*  738
AhFRO8.1    TCGSNIGVLVCGPETLKESVAAACRKQSNCFKFGGGKRTEPCFTFHSLNFTL*  707
AhFRO8.2    TCGSNIGVLVCGPESMKESVAAACRKESNCFKFGGGKRTEPCFAFHSLNFTL*  707
AiFRO2      LKGSNAGVLVSGPKEMRQEVAAVCSSNL-----------AENLHFESFSFNW*  727
AiFRO7      WGHVDVGVLVCGPPTLQESVANEIRAHS-----MIRQRHYPIFHFHSHSFDL*  739
AiFRO8      TCGSNIGVLVCGPESMKESVAAACRKESNCFKFGGGKRTEPCFAFHSLNFTL*  707
```

Figure S2. Complete multiple sequence alignment of the FRO proteins from peanut, *A. duranensis*, and *A. ipaensis* with AtFRO2 as a reference. The three short sequence proteins (AhFRO2.4, AdFRO2.2 and AdFRO2.3) were not included. Putative transmembrane (TM) segments are numbered consecutively and marked in red box. Identical residues are highlighted in black and homologous residues in gray, while the typical conserved domains are highlighted in yellow color.
